# The Treatment of Migraine

**Published:** 1894-12-22

**Authors:** Wheelton Hind

**Affiliations:** Assistant Surgeon North Staffordshire Infirmary


### THE TREATMENT OF MIGRAINE.

By Wheelton Hind, M.D., F.R.C.S., Assistant Surgeon North Staffordshire Infirmary.

The treatment of migraine, commonly known as "the sick headache," must fall to the lot of every practitioner, and from the side of the patient any alleviation is most welcome.

Migraine seems to be a most curious complaint, attacking in various forms those who are its victims. The acuteness of the pain in the head, the excessive vomiting and the complete prostration, resulting from a severe attack, are so pronounced that it is difficult to conceive that the disease is merely one of disordered function.

I have unfortunately been able to study the complaint from two sides, that of the patient ever since I was a child, and for the last fifteen years or more from the physician's point of view. The rapid convalescence and freedom from recurrence for varying periods forbid one to presume that any gross lesion is at the bottom of migrainic attacks, but I have noted in my own case that many of the attacks were due to certain definite causes.

Without going deeply into the symptoms of the trouble, I will merely point out that there are two forms of migraine, one much less severe than the other, and which has not the definite stages of the severe attack. This form is characterised by a dull headache, which does not incapacitate for work to any extent or hinder thought, though it may require a greater effort to concentrate one's ideas, and perhaps the process of thought is somewhat slower. This form often comes on on rising, and remains till after dinner or tea, a full meal generally ending the trouble.

The acute form has certain definite stages, premonitory, where the symptoms are generally visual, blurring of the field of vision, or a rapid movement around an object, followed by a gradual onset of headache and gastric symptoms, with vomiting. The headache rapidly becomes very acute, so that it is necessary to lie down, and is followed after a greater or less period of discomfort by sleep, from which the patient wakes convalescent, but with a peculiar wooden or empty feeling in the head.

Now as to causation. I have found that my attacks were due to one of two causes, either overwork plus the absence of a meal, or the eating of certain definite articles which acted as a poison, which in my case have been of the nature of fruit. Blackberries, strawberries, raspberries, plums, greengages, if eaten raw, *i.e.*, without sugar, would always precipitate an attack without fail, although till five years ago only the blackberries had that effect. Trying to unravel the meaning of all this I came to the conclusion that there were two prime causes of my migrainic attacks, either a starvation of the brain centres (for, from the symptoms, I think there is no doubt that migraine is cerebral always not gastric, the stomach being affected reflexly along the vagi nerves), or the circulation in the vessels of the brain of a poison which is rapidly eliminated. I therefore determined to see how far treatment adapted to this view would affect the symptoms.

I always found that whenever I eat the fruits otherwise poisonous with sugar I had no attack, the sugar acting in some way as an antidote, or producing more food for the brain centres; I therefore tried a more sugary diet, taking it in the form of preserves, honey and id omne genus, at least three times daily, and have found not only in myself but in many patients that the attacks became much fewer and less violent. Cod-liver oil I also prescribed with great benefit, and at the same time I insisted on the following prescription being taken for a month or so daily: Ac. nitrohydrochl. dil., ʒi. gs.; liq. arsenici hydrochl., ʒ ss.; liq. strychninæ, ʒ ss.; syrupi lemons, ℥ ss.; aq. ad. ℥viii., ℥i. t. die. post. cib.

In my own case I have only had three abortive attacks in as many years, and many of my patients have had intermissions of over a year.

With regard to the immediate treatment of the attack, I place most reliance on antifebrin in five-grain doses, to which is added caffein-citras, given as a powder every four hours. I always found antipyrine made me worse, prolonging the attack and bringing on rapid relapses.

The hunger that is often experienced towards the end of the attack points out the necessity of the body for some food, and the rapid cure of the slighter forms of attack by a good meal indicate that cerebral starvation plays a great part in the causation of migraine.

I do not suppose that in every case exactly the same dietetic causes will obtain, but I think it probable that with care and attention the patient and the medical man may be able, in the majority of cases, to find out some special article of diet which induces an attack when eaten under certain conditions. Doubtless there are other ways in which an attack is induced, uch as great mental excitements, bright lights, concerts, theatres, but here we only see again cerebral starvation produced by a more rapid expenditure of cerebral force very frequently combined with the postponement of an accustomed meal. Such causes may often be rendered harmless by judicious refreshment. Although many writers have hinted at a close connection between migraine and epilepsy I have never seen any connection between them. Migraine has a tendency to disappear with advance in life, never leads to insanity, unconsciousness, or gross cerebral changes. The symptoms of the two complaints are totally different, the only points in common being that attacks are often nocturnal and there is some head affection. Migraine is quite harmless as far as life is concerned, and most victims are wont to accept the attacks as a necessary evil, but much can be done to ward off and also to alleviate the onslaughts of this troublesome and distressing malady on the lines which I have briefly indicated.

